# Adjuvant Radiotherapy Versus Surveillance for Grade 2 Intracranial Meningiomas: A Multi-Institutional Propensity Score-Matched Study

**DOI:** 10.3389/fonc.2022.877244

**Published:** 2022-07-01

**Authors:** Hwa Kyung Byun, Won Ick Chang, Joo Ho Lee, Chul-Kee Park, In Ah Kim, Chae-Yong Kim, Jaeho Cho, Eui Hyun Kim, Jong Hee Chang, Seok-Gu Kang, Ju Hyung Moon, Sang Hyung Lee, Jason Joon Bock Lee, Il Han Kim, Chang-Ok Suh, Chan Woo Wee, Hong In Yoon

**Affiliations:** ^1^Department of Radiation Oncology, Yonsei Cancer Center, Yonsei University College of Medicine, Seoul, South Korea; ^2^Department of Radiation Oncology, Seoul National University Hospital, Seoul National University College of Medicine, Seoul, South Korea; ^3^Department of Neurosurgery, Seoul National University Hospital, Seoul National University College of Medicine, Seoul, South Korea; ^4^Department of Radiation Oncology, Seoul National University Bundang Hospital, Seoul National University College of Medicine, Seongnam, South Korea; ^5^Department of Neurosurgery, Seoul National University Bundang Hospital, Seoul National University College of Medicine, Seongnam, South Korea; ^6^Department of Neurosurgery, Severance Hospital, Yonsei University College of Medicine, Seoul, South Korea; ^7^Department of Neurosurgery, Seoul Metropolitan Government - Seoul National University (SMG-SNU) Boramae Medical Center, Seoul, South Korea; ^8^Department of Radiation Oncology, Kangbuk Samsung Hospital, Sungkyunkwan University School of Medicine, Seoul, South Korea; ^9^Department of Radiation Oncology, Bundang CHA Medical Center, CHA University, Seongnam, South Korea; ^10^Department of Radiation Oncology, Seoul Metropolitan Government - Seoul National University (SMG-SNU) Boramae Medical Center, Seoul, South Korea; ^11^Department of Radiation Oncology, Seoul National University College of Medicine, Seoul, South Korea

**Keywords:** adjuvant radiotherapy, surveillance, intracranial meningioma, surgical resection, propensity score matching

## Abstract

**Purpose:**

We aimed to compare the outcomes of adjuvant radiotherapy (ART) and surveillance in patients with grade 2 meningiomas (MNG2) who underwent surgical resection.

**Materials and Methods:**

Data from four hospitals, in which patients aged ≥18 years underwent Simpson grade 1−4 surgical resection for newly diagnosed MNG2 between 1998 and 2018, were examined in this multicenter retrospective cohort study. Patients receiving ART with conventional fractionation were compared with those undergoing surveillance. Progression-free survival (PFS), progression/recurrence (P/R) were evaluated.

**Results:**

This study included 518 patients, 158 of whom received ART. The median follow-up duration was 64.9 months. In the total cohort, ART was independently associated with significantly improved PFS (HR, 0.35; 95% CI, 0.23–0.55; P<0.001) and P/R (HR, 0.30; 95% CI, 0.18–0.48; P<0.001). In the propensity score-matched cohort (n=143 in each group), the 5-year PFS rates were 80.8% and 57.7% (P=0.004), and the 5-year P/R rates were 16.5% and 40.0% (P=0.002) in the ART and surveillance groups, respectively. After gross total resection, the 5-year PFS (85.0% vs. 64.7%; P=0.020) and P/R rates (15.2% vs. 32.0%; P=0.035) were significantly better in the ART group than in the surveillance group. A model for P/R was developed using recursive partitioning analysis with surgical extent, tumor size, and Ki-67 index. ART reduced the risk of P/R in the low- (P=0.069), intermediate- (P=0.044), and high-risk groups (P<0.001). Local control was also significantly enhanced by ART among all the risk groups (all P<0.05).

**Conclusions:**

ART significantly improved PFS and P/R in patients with MNG2, irrespective of the surgical extent, and can be recommended after gross total resection. A prognostic model may guide decision-making for the use of ART.

## Introduction

Meningioma is the most common type of intracranial tumor, accounting for 35% of all intracranial tumors diagnosed (1). World Health Organization grade is related to tumor behavior and prognosis as follows (2): grade 1, benign; grade 2, atypical; and grade 3, malignant. The recurrence rates for benign, atypical, and malignant meningioma are approximately 7%–20%, 30%–40%, and 50%–80%, respectively (3). A vast majority of meningiomas are grade 1, constituting approximately 80–95% of all the meningiomas. Meningiomas of grade 2 (MNG2) and grade 3 are relatively rare, constituting approximately 4%–15% and 1%–3%, respectively (3, 4).

Surgical resection remains the first-line treatment for MNG2 and grade 3 meningiomas (5). Adjuvant radiotherapy (ART) has been presented to improve the treatment outcomes in patients with grade 3 meningiomas; however, there is no clear consensus regarding the use of ART in patients with MNG2. Currently, the cooperative group randomized controlled trials, including the ROAM/EORTC-1308 (6) and NRG Oncology BN003 trials (7), are being held to compare early ART with active surveillance in MNG2. The retrospective studies should guide the clinical practices and elucidate the role of ART until data from these trials develop. However, the results of small retrospective series are conflicting. ART is generally indicated following subtotal resection (STR) of MNG2; nonetheless, the role of ART following gross total resection (GTR) remains controversial (8–14).

Therefore, we aimed to compare the treatment outcomes between ART and surveillance following surgical resection of MNG2 in a large multicenter retrospective cohort. Furthermore, we sought to identify which patients may benefit most from ART, to aid in clinical decision-making.

## Materials and Methods

### Cohort Development

We collected data from four hospitals in which the patients were treated. The inclusion criteria were as follows: i) newly diagnosed intracranial MNG2 according to the WHO 2016 classification (15), ii) diagnosed between 1998 and 2018, iii) surgically resected, and iv) aged ≥18 years. By reviewing the pathology reports of patients diagnosed before 2016, patients were re-classified according to the WHO 2016 classification. The exclusion criteria were as follows: i) history of any other brain tumor; ii) neurofibromatosis type 2; iii) optic nerve sheath meningioma; iv) multiple meningioma; v) history of brain radiotherapy; vi) history of other malignancy within 5 years, excluding *in situ* tumors of the uterine cervix, *in situ* tumors of the breast, differentiated thyroid cancer, and basal cell carcinoma; and vii) ART with stereotactic radiosurgery. The inclusion and exclusion criteria were primarily based on those utilized in the ROAM/EORTC-1308 trial (6). In total, 518 patients were assessed. This study was approved by the institutional review boards of each participating hospital.

### Treatment and Follow-Up

All the patients underwent surgical resection as an initial treatment, following diagnosis. The extent of resection was determined based on the surgeons’ descriptions. In 76.3% of the cases, the postsurgical magnetic resonance imaging (MRI) findings were also used for evaluation. The extent of resection was dichotomized into GTR (Simpson grade 1–3) versus STR (Simpson grade 4). Patients undergoing biopsy (Simpson grade 5) were not included. Radiotherapy performed within a year following surgery without any evidence of disease progression or recurrence (P/R) was considered to be ART. ART was administered within 3 months following surgery in most patients (n=133, 84.2%). Two-dimensional, three-dimensional, and intensity-modulated radiotherapy were used in 11 (7.0%), 40 (25.3%), and 107 (67.7%) patients, respectively. The median ART dose was 59.4 Gy in 33 fractions (interquartile range [IQR], 54–60 Gy). Target volume was defined as a postsurgical tumor bed or residual gross tumor in addition to 1.5- to 2-cm margin to the meninges and 0.5- to 1-cm margin to the brain parenchyma, and an additional margin of 0.3- to 0.5-cm to the planning target volume. Follow-up MRI was performed every 6–12 months for 5 years.

### Statistical Analysis

The follow-up was calculated from the date of initial surgery. Local failure was defined as recurrence within a 2-cm margin from the tumor bed. Intracranial failure other than local failure was termed as distant intracranial failure. Any failure (local, distant intracranial, or extracranial failure) was termed as progression/recurrence (P/R). Progression-free survival (PFS) was defined as the time from initial surgery to P/R, death, or the last follow-up.

The Cox proportional hazards model was used for univariable and multivariable analyses. Clinically relevant factors regarding the patient, tumor, and treatment characteristics were included in the model. Factors with a P-value<0.1 in the univariable analyses were included in the multivariable analyses. Propensity score matching between the ART and surveillance groups was performed using a 1:1 nearest-neighbor (greedy-type) matching and a caliper width equal to 0.2 of the standard deviation of the logit measured using the R package ‘‘MatchIt” (16). The matching covariates included the age, sex, Eastern Cooperative Oncology Group (ECOG) performance status, tumor size, extent of resection, use of postoperative MRI, pathologic type, tumor location, brain invasion, bone invasion, and Ki-67 proliferation index. The standardized mean difference was used to evaluate the balance of covariate distribution between the two groups. Additionally, the balances in the covariates were assessed using the McNemar’s tests and Wilcoxon signed-rank tests for categorical variables and continuous variables, respectively. Recursive-partitioning analysis (RPA) model was developed for the patients in the surveillance group to identify the factors that were the most influential for P/R using the R package “rpart” (17). RPA was conducted with factors including the age, sex, ECOG performance status, tumor size, extent of resection, use of postoperative MRI, pathologic type, tumor location, brain invasion, bone invasion, and Ki-67 proliferation index. The performance of the RPA model was evaluated using area under curve (AUC). For internal validation of the model, bootstrap resampling (1,000 iterations) was performed. A two-sided P-value <0.05 was considered significant. Statistical analyses were performed using R software version 4.0.3 (R Foundation for Statistical Computing, Vienna, Austria) and SPSS software version 25.0 (IBM Inc., Armonk, NY, USA).

## Results

### Patient Characteristics

Of the 518 patients, 158 (30.5%) and 360 (69.5%) patients underwent ART and surveillance, respectively (**Table 1**). The ART group exhibited a significantly larger tumor size (median, 5 cm vs. 4.5 cm, P<0.001), more frequent STR (30.4% vs. 14.7%, P<0.001), and higher Ki-67 levels (median, 5.0% vs. 4.4%, P=0.027) compared with the surveillance group. The median time from initial surgery to ART was 1.3 months (IQR, 1.0–2.1 months).

**Table 1:** Baseline characteristics in the entire and matched cohorts.

	Entire cohort*				Matched cohort^†^			
	ART (N=158)	Surveillance (N=360)	*P*	SMD	ART (N=143)	Surveillance (N=143)	*P*	SMD
Age (median, range)	54.2 (45.2–61.3)	53.9 (45–64.9)	0.347	-0.119	55.1 (46.2–61.7)	52.4 (43.7–63.2)	0.866	0.009
Sex			0.641	-0.018			>0.999	0.014
Male	64 (40.5)	138 (38.3)			57 (39.9)	58 (40.6)		
Female	94 (59.5)	222 (61.7)			86 (60.1)	85 (59.4)		
ECOG performance status			<0.001	0.664			0.001	-0.040
0	24 (15.2)	183 (50.8)			22 (15.4)	39 (27.3)		
1	117 (74.1)	135 (37.5)			106 (74.1)	75 (52.4)		
2	16 (10.1)	33 (9.2)			14 (9.8)	22 (15.4)		
3	1 (0.6)	8 (2.2)			1 (0.7)	7 (4.9)		
4	0 (0)	1 (0.3)			0 (0)	0 (0)		
Tumor size, cm	5 (4–6.1)	4.5 (3.3–5.7)	<0.001	0.377	5.1 (4–6.2)	4.8 (3.8–6.1)	0.368	0.122
Extent of resection			<0.001	0.334			0.268	0.122
Gross total resection	110 (69.6)	307 (85.3)			101 (70.6)	109 (76.2)		
Subtotal resection	48 (30.4)	53 (14.7)			42 (29.4)	34 (23.8)		
Postoperative MRI			<0.001	0.557			0.607	-0.071
No	20 (12.7)	103 (28.6)			14 (9.8)	11 (7.7)		
Yes	138 (87.3)	257 (71.4)			129 (90.2)	132 (92.3)		
Pathology			0.663	0.013			NA	0.081
Atypical	152 (96.2)	347 (96.4)			137 (95.8)	140 (97.9)		
Clear cell	2 (1.3)	2 (0.6)			2 (1.4)	0 (0)		
Chordoid	4 (2.5)	11 (3.1)			4 (2.8)	3 (2.1)		
Location			0.351	-0.011			0.578	0.024
Convexity	76 (48.1)	174 (48.3)			65 (45.5)	70 (49)		
Falx/Parasagittal/Tentorium	39 (24.7)	84 (23.3)			37 (25.9)	35 (24.5)		
Skull base	40 (25.3)	83 (23.1)			38 (26.6)	30 (21)		
Ventricle	3 (1.9)	19 (5.3)			3 (2.1)	8 (5.6)		
Brain invasion			0.865	-0.053			0.694	-0.065
No	117 (74.1)	264 (73.3)			108 (75.5)	104 (72.7)		
Yes	41 (25.9)	96 (26.7)			35 (24.5)	39 (27.3)		
Bone invasion			0.438	0.029			0.678	0.071
No	141 (89.2)	329 (91.4)			129 (90.2)	132 (92.3)		
Yes	17 (10.8)	31 (8.6)			14 (9.8)	11 (7.7)		
Ki-67 (%)	5.0 (3–9)	4.4 (2.5–7.5)	0.027	0.111	5 (3–9)	5 (2.9–10)	0.764	-0.050

Data are presented as the median (IQR) or n (%).

ART, adjuvant radiotherapy; ECOG, Eastern Cooperative Oncology Group; MRI, magnetic resonance imaging.

*Before matching, chi-square and Wilcoxon rank-sum tests were used to analyze categorical variables and continuous variables, respectively.

^†^After matching, the standardized mean difference (SMD) was used to evaluate the balance of the covariate distribution between two groups. McNemar’s tests and Wilcoxon signed-rank tests were used to analyze categorical variables and continuous variables, respectively. NA, not applicable.

### Treatment Outcomes in the Entire Cohort

The median duration of follow-up were 64.9 months (IQR, 40.4–101.2 months), 56.8 months (IQR, 39.4–85.4 months), and 67.5 months (IQR, 41.2–108.4 months) in all the patients, the ART group, and the surveillance group, respectively. During the follow-up, 133 patients (25.7%) experienced P/R at 3.1–167.0 months (median, 23.8 months) following surgery and 49 patients (9.5%) died at 3.1–253.3 months (median, 55.1 months) following surgery. In the ART group, 29 patients (18.4%) experienced P/R with 22 (13.9%) with local failure, five (3.2%) with distant intracranial failure, and two (1.3%) with extracranial failure at the first failure time (**Table A.1**). In the surveillance group, 104 patients (28.9%) experienced P/R with 98 (27.2%) with local failure, four (1.1%) with distant intracranial failure, and two (0.6%) with both local and distant intracranial failure at the first failure time.

**Figure 1:**
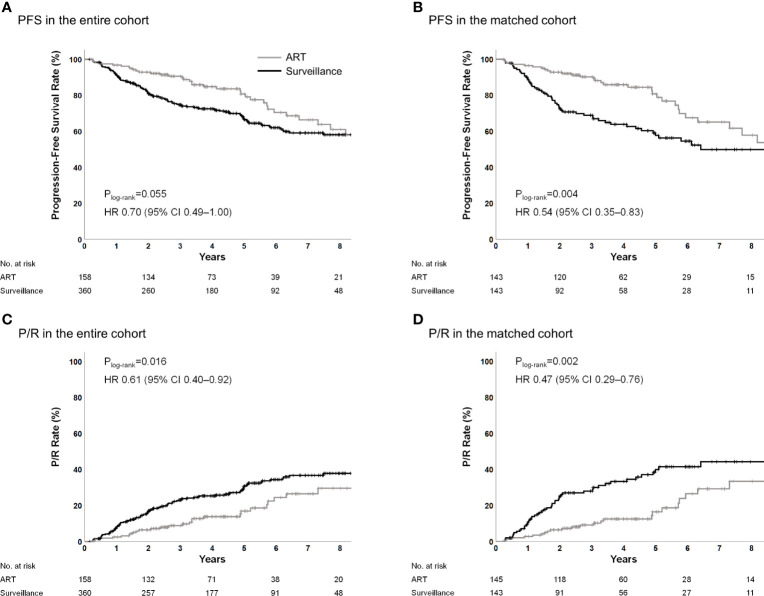
PFS and P/R in the entire and propensity score-matched cohorts. ART, adjuvant radiotherapy; PFS, progression-free survival; P/R, progression/recurrence.

The 5-year PFS rates were 80.7% in the ART group and 66.6% in the surveillance group (P_log-rank_=0.055; hazard ratio [HR], 0.70; 95% CI, 0.49–1.00; P=0.056) (**Figure 1A**). The 5-year P/R rates were 17.0% in the ART group and 30.8% in the surveillance group (P_log-rank_=0.016; HR, 0.61; 95% CI, 0.40–0.92; P=0.018) (**Figure 1C**). The 5-year local failure rates were 12.0% in the ART group and 30.2% in the surveillance group (P_log-rank_=0.001; HR, 0.46; 95% CI, 0.29–0.74; P<0.001) (**Figure A.1A**). In the multivariable analysis, older age, larger tumor size, and STR were the unfavorable prognostic factors for PFS; however, ART (HR, 0.35; 95% CI, 0.23–0.55; P<0.001) was a significantly favorable factor (**Table 2**). In the multivariable analysis, larger tumor size and STR were unfavorable prognostic factors for P/R; however, ART (HR, 0.30; 95% CI, 0.18–0.48; P<0.001) was a significantly favorable factor. ART was also independently associated with reduced local failure (HR, 0.21; 95% CI, 0.12–0.35; P<0.001).

**Table 2:** Prognostic factors for PFS and P/R in the entire cohort.

	Univariable analysis		Multivariable analysis	
	HR (95% CI)	P	HR (95% CI)	P
For PFS				
Age (per 1-year increase)	1.03 (1.02–1.04)	<0.001	1.02 (1.01–1.04)	<0.001
Female (vs. Male)	0.68 (0.50–0.93)	0.014	0.86 (0.62–1.21)	0.390
ECOG 2–4 (vs. 0–1)	1.48 (0.98–2.22)	0.060	1.07 (0.67–1.71)	0.767
Size (per 1-cm increase)	1.19 (1.11–1.26)	<0.001	1.29 (1.16–1.42)	<0.001
Subtotal resection (vs. gross total resection)	2.78 (2.01–3.85)	<0.001	3.30 (2.26–4.83)	<0.001
Clear cell or chordoid pathology (vs. atypical)	1.11 (0.52–2.37)	0.785		
Non-convexity location (vs. convexity location)	1.23 (0.90–1.68)	0.202		
Brain invasion (vs. No)	1.24 (0.89–1.73)	0.207		
Bone invasion (vs. No)	2.06 (1.36–3.15)	0.001	1.11 (0.68–1.82)	0.677
Ki-67 (per 1% increase)	1.03 (1.01–1.06)	0.017	1.02 (0.99–1.05)	0.178
ART (vs. surveillance)	0.70 (0.49–1.00)	0.056	0.35 (0.23–0.55)	<0.001
For P/R				
Age (per 1-year increase)	1.02 (1.00–1.03)	0.014	1.01 (1.00–1.03)	0.070
Female (vs. Male)	0.80 (0.57–1.13)	0.200		
ECOG 2–4 (vs. 0–1)	1.21 (0.75–1.98)	0.435		
Size (per 1-cm increase)	1.18 (1.11–1.27)	<0.001	1.33 (1.19–1.48)	<0.001
Subtotal resection (vs. gross total resection)	2.89 (2.03–4.11)	<0.001	3.63 (2.43–5.43)	<0.001
Clear cell or chordoid pathology (vs. atypical)	1.36 (0.64–2.92)	0.425		
Non-convexity location (vs. convexity location)	1.27 (0.90–1.79)	0.168		
Brain invasion (vs. No)	1.25 (0.87–1.80)	0.234		
Bone invasion (vs. No)	2.12 (1.34–3.35)	0.001	1.14 (0.68–1.89)	0.621
Ki-67 (per 1% increase)	1.03 (1.00–1.07)	0.041	1.02 (0.99–1.06)	0.170
ART (vs. surveillance)	0.61 (0.40–0.92)	0.018	0.30 (0.18–0.48)	<0.001

ART, adjuvant radiotherapy; ECOG, Eastern Cooperative Oncology Group; PFS, progression-free survival; P/R, progression/recurrence; HR: hazard ratio; CI, confidence interval.

### Treatment Outcomes in the Propensity Score-Matched Dataset

Following the propensity score matching, the ART and surveillance groups included 143 patients, each with all the characteristics being well matched (**Table 1**). The matching resulted in a reduction of the standardized mean difference below 20% (0.2) for all the covariates. There was no significant difference observed in the covariates of the groups, except for the ECOG performance status.

In the matched cohort, the 5-year PFS rates were 80.8% and 57.7% for the ART and surveillance groups, respectively (P_log-rank_=0.004; HR, 0.54; 95% CI, 0.35–0.83; P=0.005) (**Figure 1B**). The 5-year P/R rates were 16.5% and 40.0% for the ART group and surveillance groups, respectively (P_log-rank_=0.002; HR, 0.47; 95% CI, 0.29–0.76; P=0.002) (**Figure 1D**). The 5-year local failure rates were 12.0% in the ART group and 39.2% in the surveillance group (P_log-rank_<0.001; HR, 0.36; 95% CI, 0.22–0.61; P<0.001) (**Figure A.1B**).

**Figure 2:**
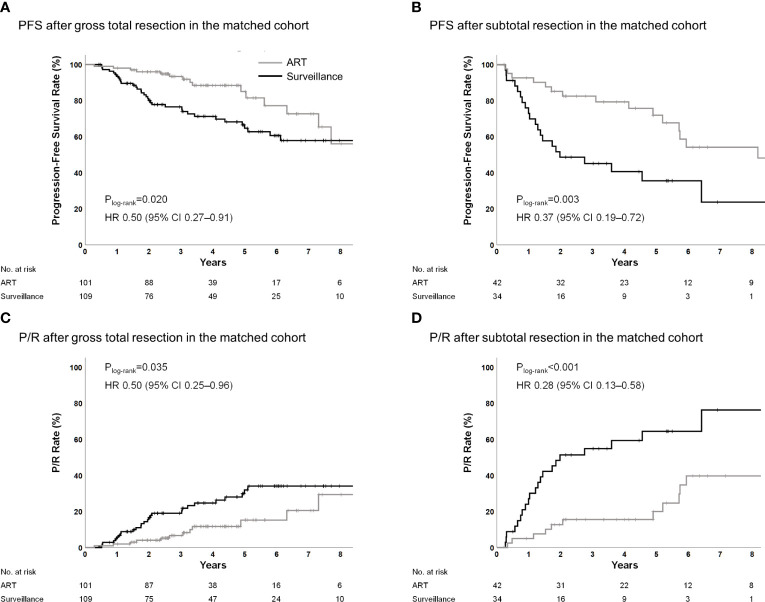
PFS and P/R for ART according to surgical extent in the propensity score-matched cohorts. ART, adjuvant radiotherapy; PFS, progression-free survival; P/R, progression/recurrence.

Among patients who had undergone GTR, the 5-year PFS rates were 85.0% and 64.7% (P_log-rank_=0.020; HR, 0.50; 95% CI, 0.27–0.91; P=0.023) (**Figure 2A**), the 5-year P/R rates were 15.2% and 32.0% (P_log-rank_=0.035; HR, 0.50; 95% CI, 0.25–0.96; P=0.038) (**Figure 2C**), and the 5-year local failure rates were 9.0% and 32.0% (P_log-rank_=0.002; HR, 0.33; 95% CI, 0.15–0.70; P=0.004) in the ART and surveillance groups, respectively (**Figure A.2A**). Among patients who had undergone STR, the 5-year PFS rates were 71.9% and 35.6% (P_log-rank_=0.003; HR 0.37; 95% CI, 0.19–0.72; P=0.004) (**Figure 2B**), the 5-year P/R rates were 20.0% vs. 64.4% (P_log-rank_<0.001; HR; 0.28; 95% CI, 0.13–0.58; P=0.001) (**Figure 2D**), and the 5-year local failure rates were 17.4% and 61.6% (P_log-rank_<0.001; HR, 0.24; 95% CI, 0.11–0.52; P<0.001) in the ART and surveillance groups, respectively (**Figure A.2B**). Treatment outcomes according to the surgical extent in the entire cohort are shown in **Figure A.3**.

**Figure 3:**
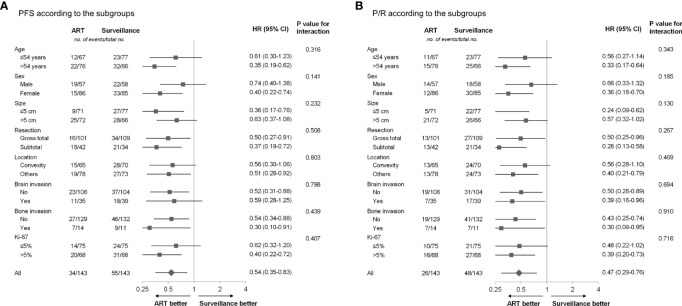
**(A)** PFS and **(B)** P/R according to subgroup in the propensity score-matched cohort. ART, adjuvant radiotherapy; PFS, progression-free survival; P/R, progression/recurrence.

Subgroup analyses based on the age, sex, tumor size, extent of resection, tumor location, brain invasion, bone invasion, and Ki-67 proliferation index were performed (**Figure 3**). The ART group observed better trends for PFS and P/R rates compared with the surveillance group in all the above-mentioned subgroup analyses.

### RPA Model for Progression/Recurrence

**Figure 4:**
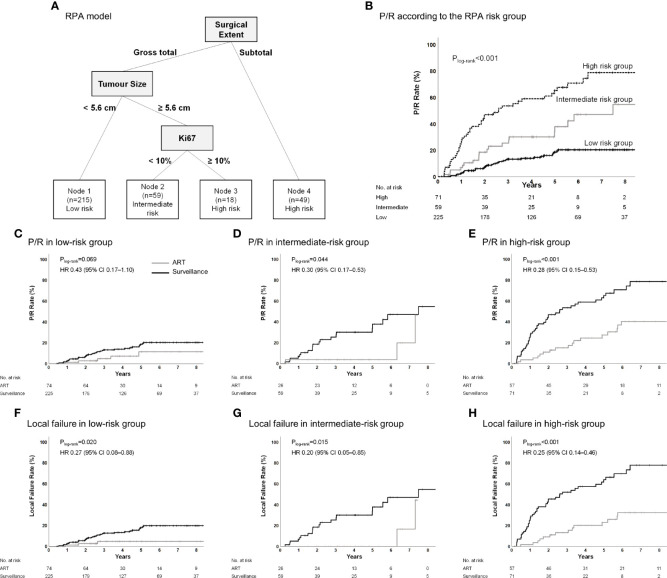
**(A)** Classification of the surveillance group into risk-of-P/R categories and **(B)** Kaplan–Meier estimates of P/R according to these categories. **(C–H)** P/R and local failure in the low-risk group, intermediate-risk group, and high-risk group according to adjuvant treatment in the entire cohort. Recursive-partitioning analysis was used to identify prognostic factors with the most influential predictive significance in a proportional-hazards model of P/R and to classify patients into categories of low-, intermediate-, or high-risk of P/R. ART, adjuvant radiotherapy; P/R, progression/recurrence.

An RPA model was generated in the surveillance group to identify patients who were at the highest risk for P/R without ART and would obtain the maximum benefit from ART. RPA revealed that the extent of resection was a major determinant of P/R. Nevertheless, this model demonstrated a high risk of P/R even after the dependence of GTR on the tumor size and Ki-67 index. The RPA model classified patients into three risk groups (**Figure 4A**). The 5-year P/R rates in the low-, intermediate-, and high-risk groups were 18.6%, 37.9%, and 65.3%, respectively (P_log-rank_<0.001) (**Figure 4B**). Further, the patients in the ART group were reincluded and classified into the respective risk groups to determine whether ART reduces P/R and local failure in each risk group. ART significantly reduced P/R in the intermediate- (P_log-rank_=0.044) and high-risk groups (P_log-rank_<0.001); however, there was a trend for reduced P/R in the low-risk group (P_log-rank_=0.069) (**Figures 4C–E**). ART significantly reduced local failure in all the risk groups (all P_log-rank_<0.05) (**Figures 4F–H**). ART was an independent prognostic factor for P/R in the high-risk groups (**Table A.2**) and for local failure in all the groups (**Table A.3**). The observed AUC of the RPA model was 0.726 (95% CI, 0.668–0.784), and the mean AUC using 1,000 bootstrap samples was 0.726 (95% CI, 0.668–0.785).

## Discussion

In this multicenter retrospective study, we compared the treatment outcomes between ART and surveillance following surgical resection of patients with MNG2. Our findings indicated that ART significantly improved PFS and decreased P/R rates compared with surveillance. Following the propensity score matching, ART resulted in significantly lower P/R and local failure rates and prolonged PFS after both STR and GTR. An RPA model for P/R was used to classify the patients in the surveillance group into three risk groups based on the surgical extent, tumor size, and the Ki-67 proliferation index. Subsequent analysis revealed that the P/R rates were significantly lower in the ART patients compared to surveillance patients in the intermediate- and high-risk groups and the local failure rates were significantly lower in ART patients compared to surveillance patients.

In accordance with the previous findings, our analysis revealed that the tumor size was a significant predictor of prognosis in patients with MNG2 (9, 10). This may be secondary to the difficulty in achieving GTR (18) and tendency for larger meningiomas to grow faster (19). Therefore, even after GTR, patients with larger tumors may be at a higher risk of P/R owing to the presence of some microscopic residuum. We observed a significant decrease in P/R when patients with larger totally resected tumors (≥5.6 cm) underwent ART; however, the benefit of ART in patients with smaller totally resected tumors (<5.6 cm) was less significant. In a study by Bruna et al. (20), a Ki-67 of ≥9.9% was associated with higher recurrence rates and a poor overall survival in patients with atypical and anaplastic meningiomas. In accordance with the previous study, our finding suggests that patients with high Ki-67 should receive adjuvant treatment.

Our analysis identified surgical extent to be an important prognostic factor in patients with MNG2 (10, 11, 13, 21). ART is generally recommended following STR (13, 14, 21, 22). In contrast, whether patients with MNG2 can benefit from ART post-GTR remains controversial, given the contradictory results of multiple small single-institutional retrospective studies (8, 13, 14, 22–29). This may be secondary to the heterogeneity in the indications for ART across institutions and selection bias, given that our ART group exhibited more unfavorable characteristics such as a larger tumor size, lesser GTR, and higher Ki-67. However, a recent meta-analysis of retrospective studies observed that ART significantly increases PFS after GTR of atypical meningiomas (30). **Table 3** shows the results of retrospective series comparing ART and surveillance and the results of prospective non-randomized studies. Although the benefit of ART after GTR is heterogeneous across the studies, our study showed a clear benefit of ART post-GTR. The result may be more robust considering that our study included more than 500 patients and the results were consistent in the multivariable model as well as in the propensity score-matched population.

**Table 3:** Summary of retrospective studies comparing adjuvant radiotherapy and surveillance after surgery for grade 2 meningiomas and prospective single arm studies showing the results of adjuvant radiotherapy.

StudyYear	Study design	Study period	Patient population	No. of patients	RT modality	Interpretation of impact of ART
Wang et al. 2017 (26)	NCDB, observational, retrospective	2009-2012	G2 MNG	2515 ART: 554, S: 1961	Unknown	ART had significantly better OS than S after STR (HR, 0.59) but not after GTR (HR 1.09).
Zeng et al. 2019 (29)	SEER, observational, retrospective	2008-2015	G2 MNG	1014 ART: 315, S: 699	Unknown	ART had significantly better OS than S after STR but not after GTR. 5-yr OS (ART vs S) GTR: 87% vs 82% STR: 78% vs 65%
Lee et al. 2020 (10)	Single center, observational, retrospective	2000-2015	G2 MNG	230 ART: 51, S: 179	SRS or fractionated RT	ART had significantly better P/R than S irrespective of surgical extent. 5-yr PFS (ART vs S) GTR: 94% vs 70% STR: 72% vs 37%
Chen et al. 2019 (22)	Single center, observational, retrospective	1993-2014	G2 MNG	182 ART: 42, S: 140	SRS or fractionated RT	ART had significantly better local control than S after GTR and STR.
Wang et al. 2019 (27)	Single center, observational, retrospective	2009-2018	G2 MNG	263 ART: 86, S: 177	Fractionated RT	ART had significantly better P/R in STR (p = 0.023) but not in GTR (p = 0.923).
Yoon et al. 2015 (28)	Single center, observational, retrospective	2000-2010	G2 MNG	158 ART: 23, S: 135	SRS or fractionated RT	ART was associated with worse PFS and OS than S.
Jenkinson et al. 2016 (24)	Single center, observational, retrospective	2001-2010	G2 MNG	133 ART: 36, S: 97	Fractionated RT	ART did not influence OS or PFS after GTR compared to S.
Weber et al., 2018 (31)	Multi-center non-randomized phase II and observational	2008-2013	G2 MNG with GTR	56 ART: 56	Fractionated RT	3-yr PFS (ART): 89%
Rogers et al., 2017 (32)	Multi-center non-randomized phase II	2009-2011	G2 MNG or recurrent benign MNG	52 ART: 52	Fractionated RT	3-yr PFS (ART): 94%
Present study 2022	Multicenter, observational, retrospective	1998-2018	G2 MNG	518 ART: 158, S: 360	Fractionated RT	ART had significantly better PFS than S irrespective of surgical extent. 5-yr PFS (ART vs S) GTR: 85% vs 65% STR: 72% vs 36%

ART, adjuvant radiotherapy; G2, grade 2; GTR, gross total resection; S, surveillance; SEER, Surveillance, Epidemiology, and End Results; SRS, stereotactic radiosurgery; STR, subtotal resection; PFS, progression-free survival; MGN, meningioma; NCDB, National Cancer Database; RT, radiotherapy.

The previous studies have attempted to establish guidelines for the adjuvant treatments with a detailed risk stratification with various clinical factors (8, 22, 33). Furthermore, there have been suggestions for molecular classification of meningioma (34–36). In this study, RPA identified three groups of P/R risk following surgery and surveillance. Our risk stratification suggests that ART can be recommended in the cases of intermediate- and high-risk MNG2. In addition, although ART exerted only a marginally significant benefit on P/R in the low-risk patients, ART reduced the risk of P/R in these patients by half. Surveillance following surgery resulted in a 5-year P/R rate of 20% in the low-risk patients. Furthermore, ART significantly reduced the risk of local failure across all the risk groups. Therefore, ART can also be considered for disease control among the low-risk patients.

Stereotactic radiosurgery has attracted the attention as an excellent alternative to external beam radiation therapy (37, 38). Although it is generally used for small residual tumors and external beam radiation therapy is preferred for MNG2 with brain and/or bone invasion (14), previous studies reported a significant portion of patients treated with adjuvant radiosurgery after STR (10, 14, 23, 39, 40) and the tumor control rate of these patients was about 60% at 5 years. After matching the patient demographics, the tumor control was similar between the two radiation modalities (39). The result of our study indicated that irradiating the whole tumor bed with additional margins lowered the P/R rate after GTR. However, as GTR is a strong prognostic factor for good prognosis, irradiating a smaller field with radiosurgery may be acceptable for patients with small tumor bed and feasible for radiosurgery

To our knowledge, this study is among the largest ones to evaluate the role of ART in patients with MNG2. Given the large sample size, we could obtain robust results with detailed analyses including multivariable analysis, propensity score matching, and RPA models. RPA aided in classifying the patients into risk groups that exhibited significant differences in prognosis, thus, aiding in the development of the therapeutic strategies for each risk group. Additionally, the results of our study are strengthened by the fact that the patients from multiple institutions with heterogenous indications for ART were merged to form a less biased cohort. Furthermore, the relatively long median follow-up duration enabled us to account for late P/R, which was identified even 5 years following surgery.

This study had several limitations owing to its retrospective nature. The differences in the patients’ characteristics suggested selection bias. However, we attempted to account for such bias using the propensity score matching and adjusted multivariable analyses. Additionally, since this study involved patients from multiple institutions, inter-institutional discrepancies in the histopathologic evaluation for the diagnostic criteria of MNG2 were inevitable. Further studies verifying our risk stratification for MNG2 treated by radiosurgery are warranted.

## Conclusions

In conclusion, our findings indicated that ART improved PFS and P/R in patients with MNG2, irrespective of the surgical extent. The RPA model for P/R may guide the clinicians in decision-making regarding ART following resection of MNG2; however, we recommend ART for all the risk groups when the goal is to reduce P/R. Further studies can validate our results and establish indications for ART.

## Data Availability Statement

The raw data supporting the conclusions of this article will be made available by the authors, without undue reservation.

## Ethics Statement

Studies involving human participants were reviewed and approved by the Institutional Review Board of Severance Hospital (IRB No. 4-2020-1052).

## Author Contributions

CWW and HIY contributed to protocol development, including literature search, study design, funding acquisition, study coordination, and quality assurance. JHL, C-KP, IAK, C-YK, JC, EHK, JHC, S-GK, JHM, SHL, JJBL, IHK, C-OS contributed to patient enrollment, treatment, follow-up, and data collection. HKB contributed to statistical analysis. HKB, WIC, CWW, HIY verified underlying data, analyzed the data, and wrote the manuscript. All authors contributed to revision of and commenting on the manuscript and approved the manuscript.

## Funding

This work was supported by the National Research Foundation of Korea (NRF) grant funded by the Korea Government (MSIT) (No. 2020R1F1A1076287) and Basic Science Research Program through the NRF funded by the Ministry of Education (No. NRF-2021R1I1A1A01059636).

## Conflict of Interest

The authors declare that the research was conducted in the absence of any commercial or financial relationships that could be construed as a potential conflict of interest.

## Publisher’s Note

All claims expressed in this article are solely those of the authors and do not necessarily represent those of their affiliated organizations, or those of the publisher, the editors and the reviewers. Any product that may be evaluated in this article, or claim that may be made by its manufacturer, is not guaranteed or endorsed by the publisher.
