# Supplementary material for: Adjuvant Radiotherapy Versus Surveillance for Grade 2 Intracranial Meningiomas: A Multi-Institutional Propensity Score-Matched Study
Source: Front Oncol. 2022 Jul 1;12:877244. doi: 10.3389/fonc.2022.877244 (PMC9283569; doi:10.3389/fonc.2022.877244)
Supplement: Supplementary file 1 [file DataSheet_1.docx]

Adjuvant Radiotherapy Versus Surveillance for Grade 2 Intracranial Meningiomas: A Multi-Institutional Propensity Score-matched Study

Supplementary Material

Figure A.1. Local failure in the entire and matched cohorts.

Figure A.2. Local failure according to treatment and surgical extent in the matched cohort.

Figure A.3. PFS, P/R, and local failure according to treatment and surgical extent in the entire cohort.

Table A.1. Pattern of first disease progression

Table A.2. Multivariable analyses of factors associated with P/R according to risk group

Table A.3. Multivariable analyses of factors associated with local failure according to risk group

# Supplementary Figures and Tables

**Figure A.1.** Local failure in the entire and matched cohorts.

Abbreviations: ART, adjuvant radiotherapy

**Figure A.2.** Local failure according to treatment and surgical extent in the matched cohort.

Abbreviation: ART, adjuvant radiotherapy

**Figure A.3.** PFS, P/R, and local failure according to treatment and surgical extent in the entire cohort.

Abbreviations: ART, adjuvant radiotherapy; PFS, progression-free survival; P/R, progression/recurrence

**Table A.1.** Pattern of first disease progression

|  | ART (N=158) | Surveillance (N=360) |
| --- | --- | --- |
| Any failure | 29 (18.4) | 104 (28.9) |
| Local failure | 22 (13.9) | 98 (27.2) |
| Distant intracranial failure | 5 (3.2) | 4 (1.1) |
| Extracranial failure | 2 (1.3) | 0 (0) |
| Local and distant intracranial failure | 0 (0) | 2 (0.6) |

Abbreviation: ART, adjuvant radiotherapy. Data are n (%).

**Table A.2.** Multivariable analyses of factors associated with P/R according to risk group

|  | Entire cohort | |  | Low-risk group | |  | Intermediate-risk group | |  | High-risk group | |
| --- | --- | --- | --- | --- | --- | --- | --- | --- | --- | --- | --- |
|  | HR (95% CI) | P |  | HR (95% CI) | P |  | HR (95% CI) | P |  | HR (95% CI) | P |
| Age (per 1-year increase) | 1.01 (1.00–1.03) | 0.070 |  | 0.99 (0.97–1.02) | 0.583 |  | 1.02 (0.99–1.06) | 0.175 |  | 1.03 (1.01–1.05) | 0.006 |
| Size (per 1-cm increase) | 1.33 (1.19–1.48) | <0.001 |  | 1.34 (0.96–1.87) | 0.085 |  | 1.01 (0.56–1.82) | 0.973 |  | 1.15 (0.97–1.36) | 0.114 |
| Subtotal resection (vs. gross total resection) | 3.63 (2.43–5.43) | <0.001 |  | NA |  |  | NA |  |  | 1.07 (0.48–2.36) | 0.871 |
| Bone invasion (vs. No) | 1.14 (0.68–1.89) | 0.621 |  | 2.00 (0.68–5.85) | 0.206 |  | 2.76 (0.75–10.18) | 0.126 |  | 0.86 (0.45–1.62) | 0.631 |
| Ki-67 (per 1% increase) | 1.02 (0.99–1.05) | 0.17 |  | 1.01 (0.95–1.08) | 0.689 |  | 0.96 (0.78–1.19) | 0.710 |  | 0.98 (0.93–1.03) | 0.465 |
| Adjuvant radiotherapy (vs. surveillance) | 0.30 (0.18–0.48) | <0.001 |  | 0.47 (0.18–1.20) | 0.115 |  | 0.35 (0.10–1.20) | 0.096 |  | 0.26 (0.14–0.48) | <0.001 |

Abbreviations: ART, adjuvant radiotherapy; P/R, progression/recurrence; HR, hazard ratio; CI, confidence interval

**Table A.3.** Multivariable analyses of factors associated with local failure according to risk group

|  | Entire cohort | |  | Low-risk group | |  | Intermediate-risk group | |  | High-risk group | |
| --- | --- | --- | --- | --- | --- | --- | --- | --- | --- | --- | --- |
|  | HR (95% CI) | P |  | HR (95% CI) | P |  | HR (95% CI) | P |  | HR (95% CI) | P |
| Age (per 1-year increase) | 1.01 (1.00–1.03) | 0.11 |  | 0.99 (0.96–1.01) | 0.349 |  | 1.02 (0.99–1.06) | 0.243 |  | 1.03 (1.01–1.05) | 0.006 |
| Size (per 1-cm increase) | 1.34 (1.20–1.50) | <0.001 |  | 1.3 (0.93–1.83) | 0.129 |  | 1.07 (0.58–1.98) | 0.833 |  | 1.14 (0.95–1.36) | 0.160 |
| Subtotal resection (vs. gross total resection) | 3.71 (2.45–5.60) | <0.001 |  | NA |  |  | NA |  |  | 1.14 (0.50–2.59) | 0.762 |
| Bone invasion (vs. No) | 1.25 (0.74–2.09) | 0.404 |  | 1.70 (0.50–5.74) | 0.394 |  | 2.97 (0.79–11.09) | 0.106 |  | 1.03 (0.54–1.95) | 0.935 |
| Ki-67 (per 1% increase) | 1.03 (1.00–1.06) | 0.085 |  | 1.02 (0.95–1.09) | 0.559 |  | 0.96 (0.77–1.20) | 0.740 |  | 0.99 (0.94–1.04) | 0.705 |
| Adjuvant radiotherapy (vs. surveillance) | 0.21 (0.12–0.35) | <0.001 |  | 0.29 (0.09–0.94) | 0.039 |  | 0.22 (0.05–0.96) | 0.044 |  | 0.21 (0.11–0.41) | <0.001 |

Abbreviations: ART, adjuvant radiotherapy; P/R, progression/recurrence; HR, hazard ratio; CI, confidence interval
